# Is Granger Causality a Viable Technique for Analyzing fMRI Data?

**DOI:** 10.1371/journal.pone.0067428

**Published:** 2013-07-04

**Authors:** Xiaotong Wen, Govindan Rangarajan, Mingzhou Ding

**Affiliations:** 1 The J. Crayton Pruitt Family Department of Biomedical Engineering, University of Florida, Gainesville, Florida, United State of America; 2 Department of Mathematics and Centre for Neuroscience, Indian Institute of Science, Bangalore, India; Hangzhou Normal University, China

## Abstract

Multivariate neural data provide the basis for assessing interactions in brain networks. Among myriad connectivity measures, Granger causality (GC) has proven to be statistically intuitive, easy to implement, and generate meaningful results. Although its application to functional MRI (fMRI) data is increasing, several factors have been identified that appear to hinder its neural interpretability: (a) latency differences in hemodynamic response function (HRF) across different brain regions, (b) low-sampling rates, and (c) noise. Recognizing that in basic and clinical neuroscience, it is often the change of a dependent variable (e.g., GC) between experimental conditions and between normal and pathology that is of interest, we address the question of whether there exist systematic relationships between GC at the fMRI level and that at the neural level. Simulated neural signals were convolved with a canonical HRF, down-sampled, and noise-added to generate simulated fMRI data. As the coupling parameters in the model were varied, fMRI GC and neural GC were calculated, and their relationship examined. Three main results were found: (1) GC following HRF convolution is a monotonically increasing function of neural GC; (2) this monotonicity can be reliably detected as a positive correlation when realistic fMRI temporal resolution and noise level were used; and (3) although the detectability of monotonicity declined due to the presence of HRF latency differences, substantial recovery of detectability occurred after correcting for latency differences. These results suggest that Granger causality is a viable technique for analyzing fMRI data when the questions are appropriately formulated.

## Introduction

Granger causality [1] is a statistical method for assessing directional influences between simultaneously recorded time series [2]–[5]. Recent work demonstrates that, when applied to electrophysiological data, the directions and magnitudes of Granger causality are interpretable in terms of the directions and magnitudes of synaptic transmissions between different neurons and brain areas [6], [7]. To what extent Granger causality can be applied to functional magnetic resonance imaging (fMRI) data is debated. There are two separate issues. First, is Granger causality applicable to fMRI data? From a statistical standpoint, the realm of applicability of Granger causality is the same as any other time-series based connectivity measures such as coherence [8] and total interdependence [9], [10]. The reason is that these measures make the same assumptions about the time series under investigation (e.g., stationarity). As time-series based connectivity measures are increasingly applied to fMRI data, Granger causality, in conjunction with other methods, can provide additional empirical characteristics and biomarkers. Second, how to interpret Granger causality effects at the hemodynamic level in neural terms? Much has been written on this topic [11]–[20]. Factors underlying the current concerns include: (a) latency variability of hemodynamic response function (HRF) across different brain regions [13], [15], [21], [22], (b) low sampling rates (e.g., TR = 2 s) [14], [15], and (c) measurement noise [23], [24]. It has been shown that these factors, individually or in combination, can adversely affect fMRI Granger causality. Despite these concerns, however, highly interpretable applications of Granger causality to fMRI data continue to appear [20]. An urgent problem is how to reconcile these divergent findings and opinions. Schippers et al. via a simulation study [25], show that at the group level there is a strong correlation between significant causal directions at the fMRI level and that at the neuronal level. The issue, however, remains far from settled. The goal of this work is to further consider this problem.

Neuronal mechanisms of cognitive operations are inferred by comparing dependent variables across experimental conditions. For example, in attention research, neuronal responses under attend versus non-attend conditions are compared, whereas in working memory research, neuronal responses under different levels of working memory load are compared. It has been suggested that rather than focusing on the magnitude of Granger causality under a single experimental condition, it is more informative to focus on how it is modulated by experimental conditions [11], [26], [27]. In this context, a natural question is: Are changes in Granger causality at the fMRI level and that at the neuronal level related? More specifically, is the strength of Granger causality estimated at the fMRI level a monotonic function of the strength of Granger causality estimated at the underlying neuronal level, when a parameter is varied? If such monotonicity holds, increase or decrease of fMRI-level Granger causality as the experimental condition is varied, can then be interpreted in terms of the corresponding increase or decrease of neuronal-level Granger causality. We addressed this question by carrying out mathematical analysis and numerical simulations. For the latter, autoregressive (AR) models were used to generate neuronal level time series data. By convolution with the HRF function, downsampling, and addition of measurement noise, such time series data were then transformed into simulated fMRI signals that mimic real fMRI recordings. Strengths of Granger causal influences at the neuronal level were systematically manipulated by changing the parameters in the AR model to simulate different experimental conditions. Functional MRI-level Granger causality is plotted against the neuronal-level Granger causality to assess their relationship.

## Methods

### Simulated Neuronal Data

We generated simulated neuronal data using a bivariate autoregressive (AR) model with model order = 1:
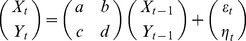
(1)


The noise covariance matrix 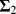 of 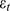 and 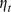 was set to be [1, 0; 0, 1], namely, 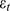 and 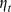 are independent and both have unit variance, and a = d = 0.8. In this model, the strength of Y→X is determined by b and the strength of X→Y by c. Two cases were studied: unidirectional coupling and bidirectional coupling. For the case of unidirectional coupling, b = 0. There were 100 experiments. For each experiment, c was chosen with equal probability between 0 and 0.8 (10 values were assigned to c in each experiment), with larger values of c corresponding to stronger X→Y. The simulated time series is 3000 s in duration and the sampling interval is assumed to be 50 ms. For the case of bidirectional coupling, there were again 100 experiments, and for each experiment, b and c were generated randomly and independently between 0 and 0.2 (10 values for b and 10 values for c in each experiment). The length of simulated time series and sampling rate were the same as in the unidirectional case. Here, the range of coupling strength variation in both cases was chosen in such a way that the resultant AR models were stationary, and different coupling strengths in the neural model simulated different experimental conditions.

### Simulated fMRI Data

**Figure 1:**
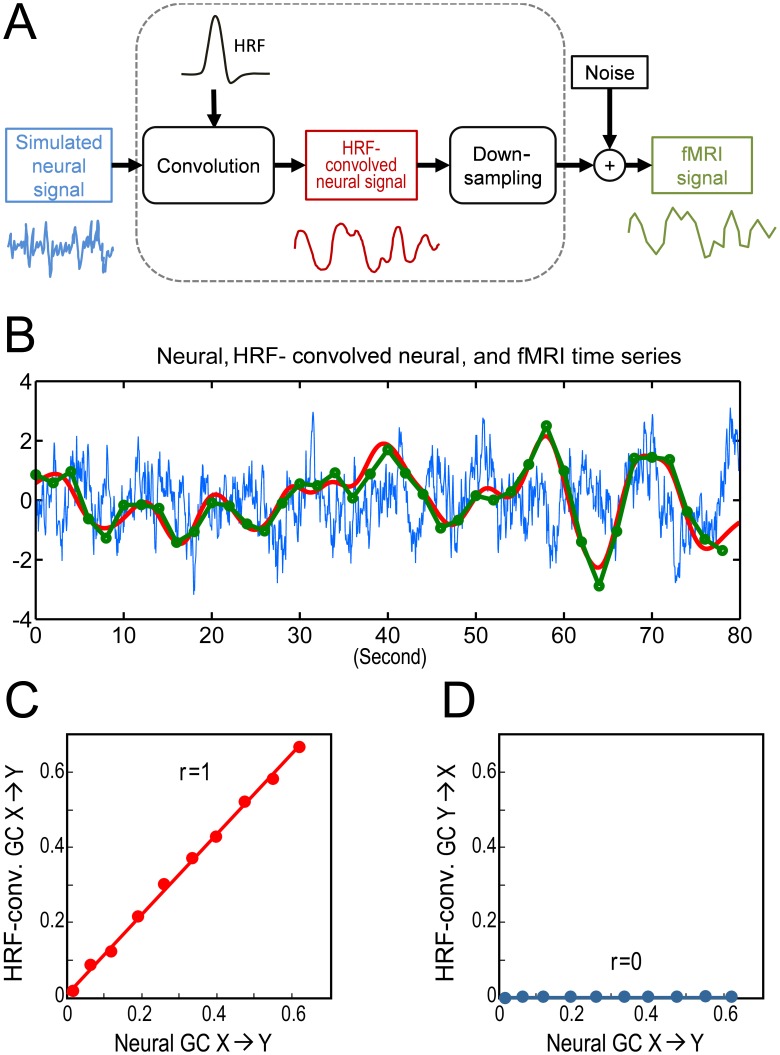
Simulated data and relation between HRF-conv. GC and neural GC. A: Flowchart illustrating the process from neural to HRF-convolved neural to fMRI data. B: An example of the process in A where the neural time series (blue) was generated using AR(1) model and was convolved with a canonical HRF to yield the HRF-convolved neural time series (red), which, after down-sampling to TR = 2 s and addition of 20% white noise (SNR = 5), became the fMRI time series (Green). C: GC for HRF-convolved neural time series as a monotonically increasing function of neural GC where the slope of fitted linear trend is close to 1. D: HRF-conv. GC in the opposite direction is zero (unidirectional coupling).

The neural time series were convolved with a canonical HRF [11], [28], [29] to yield simulated HRF-convolved neural time series. After down-sampling to commonly used temporal resolution (TR) and with addition of Gaussian noise, simulated fMRI time series were obtained. This procedure is illustrated in Figure 1A. Figure 1B shows an example of simulated original neural time series, HRF-convolved neural time series, and fMRI time series, where the level of noise for the fMRI time series is 20% (SNR = 5) and TR = 2 s.

### Theory and Estimation of Granger Causality

Consider two simultaneously recorded stationary time series 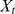
and 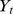. If the past of one time series can be used to facilitate the prediction of the future of the other time series, then we say there is a Granger causal influence from the former to the latter [1]. To estimate Granger causality we employ autoregressive models [2], [5], [25], [30]–[34]. Individually,
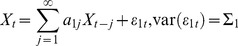
(2)

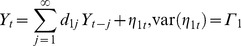
(3)


They can also be jointly represented as an unrestricted bivariate AR model:
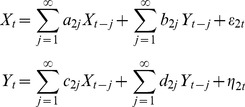
(4)


In Eq. (4), 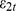 and 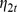 are independent over time, and their contemporaneous covariance matrix is
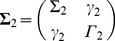
(5)


Then 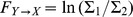 is the Granger causality from 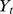 to 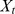
and 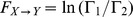 the Granger causality from 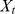 to 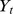 [2], [11], [30].

For practical data analysis the above infinite sums need to be truncated to sums with finite orders. For simulated neural data, the AR model order was determined to be 1 according to the Bayesian Information Criterion (BIC) [11], [35]–[37], in agreement with the order of the model in Eq. (1) used to generate the data. For the HRF-convolved neural time series before down-sampling and noise addition, the model order used was between 15 and 17. For the fMRI time series, the model order was determined to be 1 for realistic TRs between 0.5 s and 3 s, and 5 for TR = 50 ms.

### Linking Neural GC and fMRI GC

In each experiment, fMRI Granger causality (GC) was plotted against the corresponding neural GC, and the relation was assessed by Spearman rank correlation. If the correlation between fMRI GC and neural GC along the same direction (e.g., X→Y) is significantly positive then it was taken as evidence for monotonicity. In contrast, if fMRI GC in one direction (e.g., Y→X) was significantly correlated with the neural GC in the opposite direction (X→Y), the monotonicity was considered false, caused by HRF convolution, downsampling, and noise addition. Previous work has shown that in unidirectional coupling, if X→Y is nonzero but Y→X is zero at the neuronal level, HRF convolution, downsampling, and noise can lead to nonzero Y→X at the fMRI level, which is spurious [11], [18], [31]. By plotting fMRI GC against neural GC in opposite directions we examined whether such spurious effects also created false monotonicity relationships between fMRI GC and neural GC.

To quantify the simulation results, true positive rate (TPR) and false positive rate (FPR) of monotonicity detection were estimated, depending on whether fMRI GC and neural GC along the same direction and along opposite directions were significantly positively correlated. Specifically, in the unidirectional case, we defined 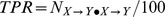, where 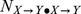 was the number of experiments in which neural X→Y and fMRI X→Y were significantly positively correlated, and 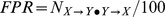, where 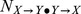 was the number of experiments in which neural X→Y and fMRI Y→X were significantly correlated. In the bidirectional case, 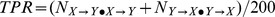, where 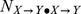 and_
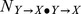
_ were the number of experiments in which neural GC and fMRI GC along the same directions were significantly positively correlated, and 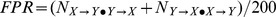, where 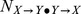 and 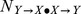 were the number of experiments in which neural GC and fMRI GC along opposite directions were significantly correlated. The detection rate (DR) of monotonicity was calculated as TPR+FPR and the true detecting rate (TDR) as TPR/DR. TPR, FPR, and TDR were examined as functions of the correlation significance threshold.

### Effects of Downsampling and Noise

TPR, FPR, and TDR were plotted as functions of different fMRI TRs, varying from 50 ms to 4 s, to study the impact of sampling rates on detecting the monotonicity relationship. Similar analysis was carried out by varying the noise level between 5% (SNR = 20) and 160% (SNR = 0.625).

### Effects of HRF Latency Variability and a Mitigation Strategy

In addition to low sampling rates and noise, another factor impacting fMRI GC analysis is HRF latency variability. We considered this issue in the case of bidirectional coupling. Across 100 experiments the HRF latency difference between X and Y followed a normal distribution with zero mean and standard deviation (SD). Varying SD from 0.2 s to 1 s, we plotted TPR, FPR, and TDR as functions of SD under slow sampling (TR = 2 s) and fast sampling (TR = 50 ms), respectively. To mitigate the adverse effects of HRF latency variability, we followed a procedure given by Chang et al. [38], based on the assumption that the HRF latency can be measured experimentally [38]–[42]: (1) up-sample the fMRI series (TR = 2 s) using the spline interpolation method to a TR of 50 ms, (2) shift the time series in such a way that the HRF latency difference is reduced to zero, (3) down-sample the latency-corrected fMRI time series to TR = 2 s, and (4) perform analysis on the latency-corrected fMRI time series as above.

## Results

### Unidirectional Coupling

In the bivariate AR(1) model in Eq. (1), the coupling strength for X→Y was between c = 0 and c = 0.8, and the coupling strength for Y→X was set at zero (b = 0). Convolving neural data with HRF yielded HRF-convolved neural signal which, after downsampling to realistic TRs (e.g., TR = 2 s) and addition of white noise (e.g., 20%, SNR = 5), became fMRI time series. This process is illustrated in Figure 1A. Subjecting the neural data and the corresponding HRF-convolved neural signal to Granger causality analysis, we found that HRF-convolved neural signal GC of X→Y (HRF-conv. X→Y for abbreviation) and neural GC of X→Y, influences along the same direction, has a linear monotonic relationship with a slope close to 1 (Figure 1C). In contrast, HRF-conv. Y→X and neural X→Y, influences along opposite directions, exhibited no systematic relationship (Figure 1D). These results suggest that the operation of HRF convolution preserves monotonicity between HRF-convolved GC and neural GC.

**Figure 2:**
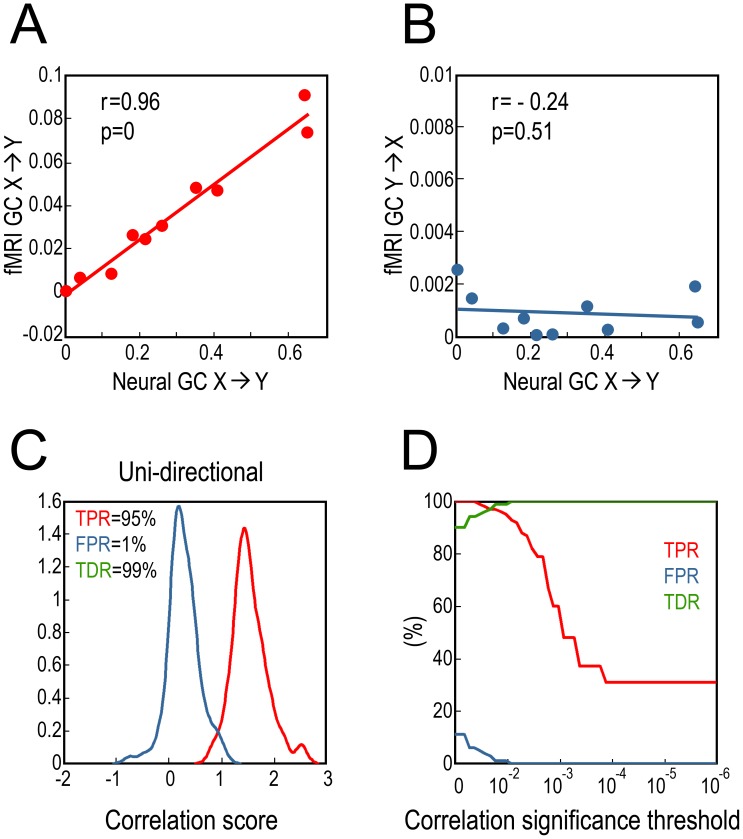
Relation between fMRI GC and neural GC (unidirectional coupling). A: A typical experiment where fMRI GC is a monotonically increasing function of neural GC. B: fMRI GC and neural GC along opposite directions are uncorrelated. C: Distributions of correlation coefficients between neural GC and fMRI GC along the same direction (red) and along opposite directions (blue). D: TPR, FPR and TDR as functions of correlation significance threshold.

For TR = 2 s and 20% noise (SNR = 5), a positive correlation between fMRI X→Y GC and neural X→Y GC was clearly seen (Figure 2A), indicating that the monotonicity between the variables can be reliably detected. No systematic relationship was found between fMRI Y→X GC and neural X→Y GC (Figure 2B). Over 100 experiments the histograms for the Fisher-transformed correlation coefficient between neural X→Y GC and fMRI X→Y GC (red) and that between neural X→Y GC and fMRI Y→X GC (blue) are shown in Figure 2C. The two distributions are well separated. TPR, FPR and TDR were plotted as functions of the correlation significance threshold (Figure 2D). If p = 0.01 is the correlation significance threshold, then in 95% of the experiments, fMRI X→Y GC was significantly and positively correlated with neural X→Y GC, whereas only in 1% of the experiments, fMRI Y→X GC was significantly correlated with neural X→Y GC. Thus, TPR = 95%, FPR = 1%, and TDR = 99%. These results indicate that even with realistic TR and noise, the monotonicity condition holds if p = 0.01 is chosen as the significance level, and we can still interpret increases in fMRI GC in terms of increases in the underlying neural GC. As significance threshold becomes more stringent, TPR gradually decreased to around 34%, but FPR became zero, leading to 100% TDR.

### Bidirectional Coupling

**Figure 3:**
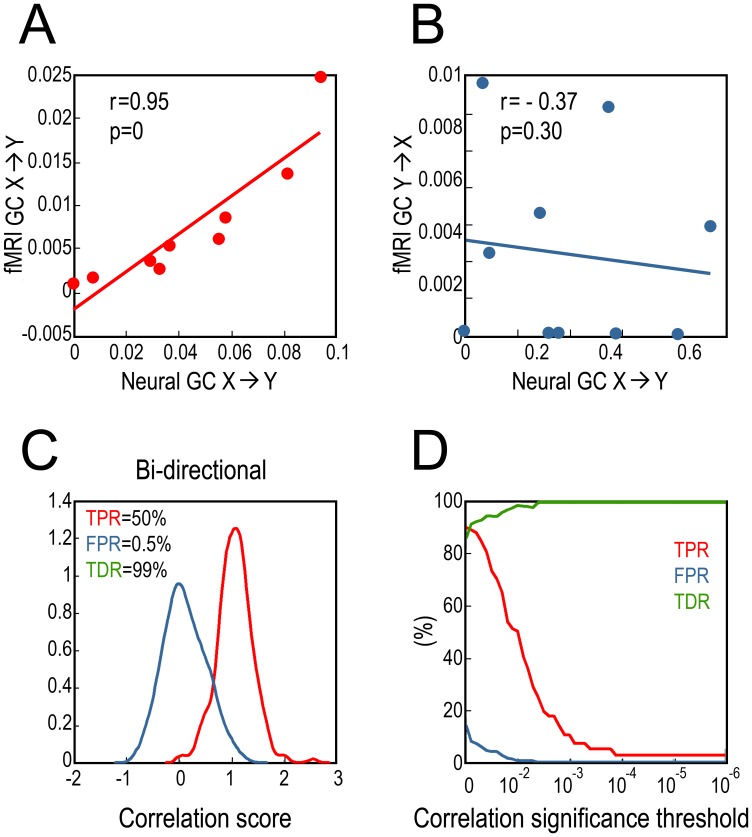
Relation between fMRI GC and neural GC (bidirectional coupling). A: A typical experiment where fMRI GC is a monotonically increasing function of neural GC. B: fMRI GC and neural GC along opposite directions are uncorrelated. C: Distributions of correlation coefficients between neural GC and fMRI GC along the same direction (red) and along the opposite directions (blue). D: TPR, FPR and TDR as functions of correlation significance threshold.

Figures 3A and 3B show the results for one experiment. Neural GC and fMRI GC along the same direction exhibit significant positive correlation whereas neural GC and fMRI GC along opposite directions are uncorrelated. Distributions of the correlation coefficient over 100 experiments are shown in Figure 3C. At the significance level of p = 0.01, TPR = 50%, FPR = 0.5%, and TDR = 99%, suggesting that the monotonic relationship between fMRI GC and neural GC can be reliably detected. Figure 3D shows TPR, FPR and TDR as functions of the significance threshold for correlation coefficient. It demonstrates that when more stringent threshold was applied, TPR decreased but remained nonzero, FPR decreased toward zero quickly, and TDR increased toward 100%.

**Figure 4:**
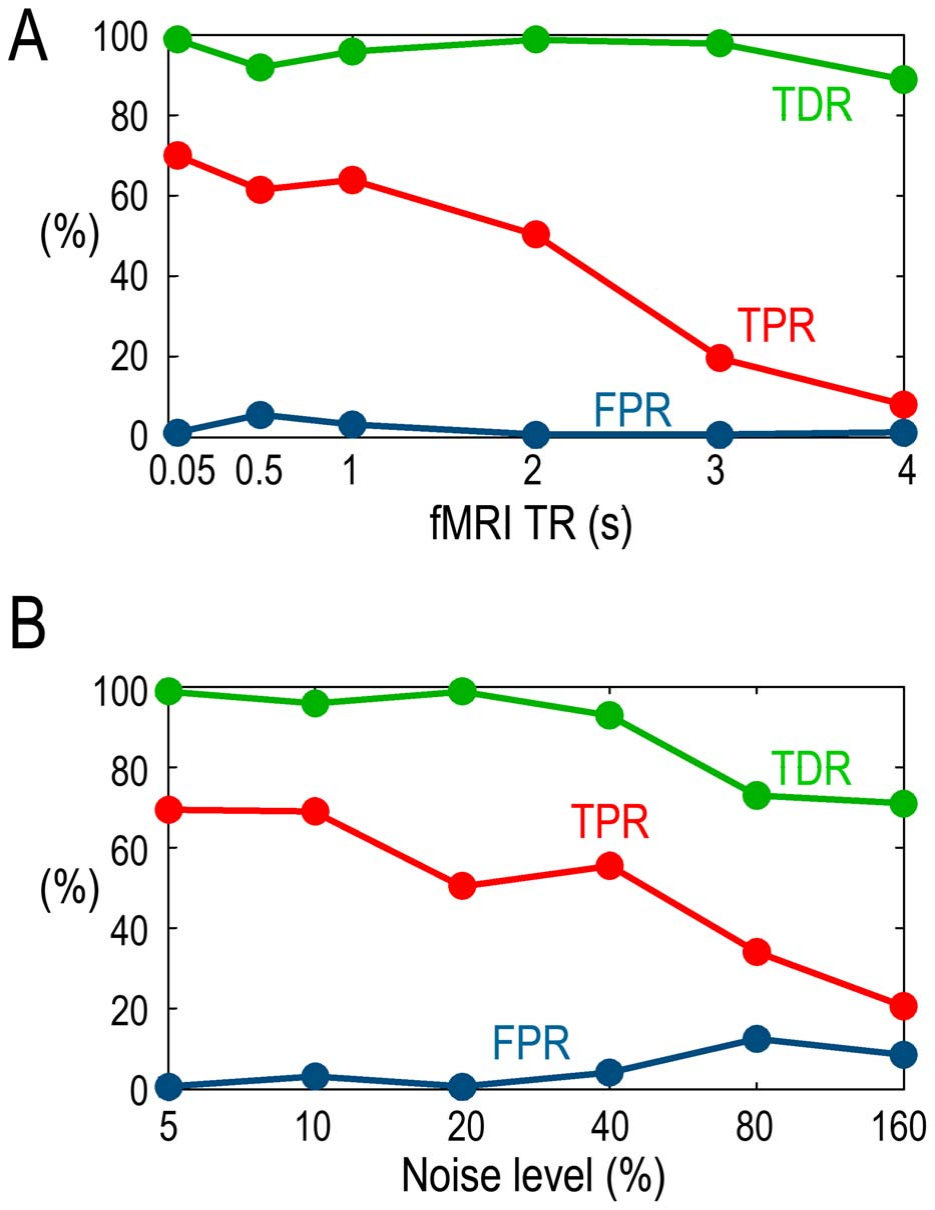
Effects of TR and noise. A: TDR, TPR and FPR as functions of fMRI TR. B: TDR, TPR and FPR as functions of the noise level.

To examine the effects of sampling rates, we plotted TPR, FPR and TDR as functions of different TRs. The noise level was fixed at 20% (SNR = 5). Although TPR decreased as TR increased, seen in Figure 4A, from TR = 50 ms to TR = 2 s, TPR remained at a reasonable level around 70% to 50%. For all the fMRI TR inspected, FPR was always below 10%, and TDR was always over 90%.

To examine the effects of noise, we plotted TPR, FPR and TDR as functions of different noise levels, with TR fixed at TR = 2 s. As seen in Figure 4B, as the noise level became higher, FPR increased and both TPR and TDR decreased. However, up to a noise level of 40% (SNR = 2.5), TPR remained at a reasonably high level between 50% to 70% and TDR over 90%. Meanwhile, FPR was below 5%.

### Effects of HRF Latency Variability

**Figure 5:**
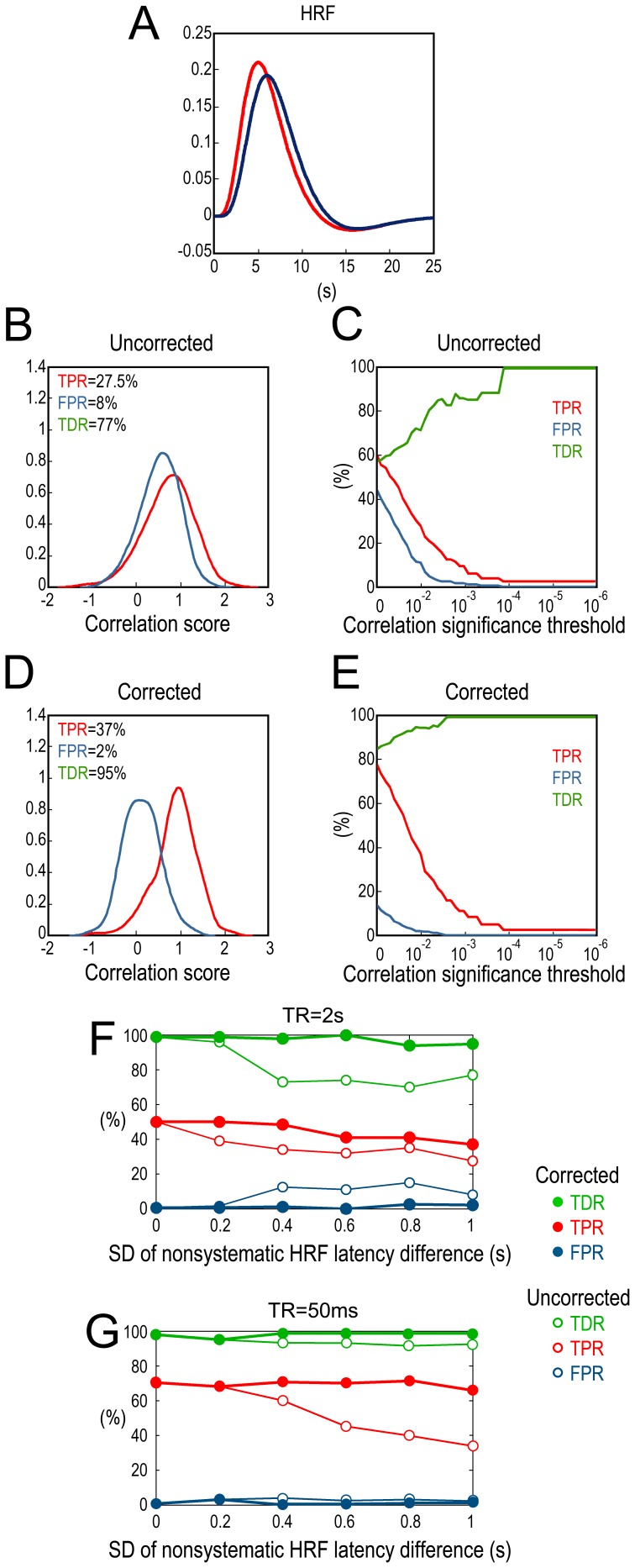
Effects of HRF latency difference. A: Two HRFs with different peak latencies. B: Distributions of correlation coefficients between neural GC and fMRI GC along the same direction (red) and along opposite directions (blue) before latency correction. C: TPR, FPR and TDR as functions of correlation significance threshold before latency correction. D: Distributions of correlation coefficients between neural GC and fMRI GC along the same direction (red) and along opposite directions (blue) after latency correction. E: TPR, FPR and TDR as functions of correlation significance threshold after latency correction. F and G: For TR = 2 s and TR = 50 ms, respectively, TPR, FPR and TDR before and after latency correction as functions of the standard deviation of the HRF latency difference distribution.

Two HRFs with different peak times are shown in Figure 5A. The HRF latency was varied by changing the parameter in the canonical HRF model that controls the time-to-peak timing. Assume that the interaction is bidirectional and latency difference in an experiment between the two areas follows a normal distribution with zero mean and a standard deviation of 0.8 s and the fMRI TR = 2 s, distributions of correlation coefficient between neural GC and fMRI GC are shown in Figure 5B where TPR is 27.5%, FPR is 8% and TDR is 77% if we set p = 0.01 as the significance threshold. TPR, FPR and TDR as functions of different significance thresholds are shown in Figure 5C. Both TPR and FPR declined but TDR increased when more stringent threshold was applied.

By applying the latency correction technique (see Method), we found that the distributions of correlation coefficient are better separated as shown in Figure 5D, where for p = 0.01, TPR improved to 37%, FPR declined to 2%, and TDR became 95%. TPR, FPR, and TDR as functions of significance threshold are shown in Figure 5E.

Further, we plotted TPR, FPR, and TDR, before and after latency correction, as functions of the standard deviation of the HRF latency difference distribution, under long and short fMRI TR (Figure 5F for TR = 2 s and Figure 5G for TR = 50 ms). The results showed that, without latency correction, when standard deviation of the latency difference increased, for TR = 2 s, TPR decreased from around 50% to 30%, FPR increased to around 10%, and TDR decreased from 99% to around 75%; for TR = 50 ms, TPR decreased from around 70% to 35%, FPR was below 4%, and TDR remained around 95%. However, following latency correction, for TR = 2 s, TPR became 40%–50%, FPR was below 2.5%, and TDR was over 95%; for TR = 50 ms, TPR became around 70%, FPR was below 3.5%, and TDR was in a range of 95%–99%.

## Discussion

Granger causality (GC) has emerged as a useful technique to evaluate directional influences in multivariate electrophysiological data. For fMRI data, however, its validity is debated. Previous simulation work has shown that convolution with HRF, low sampling rate, and addition of noise can yield spurious Granger causality [11], [18], [25], [31]. In the meantime, however, highly interpretable applications of Granger causality to fMRI data continue to appear [43], [44]. How to reconcile these divergent findings? Motivated by the observation that in cognitive neuroscience, it is often the change of a neuronal activity across experimental conditions, rather than the sheer magnitude of that activity under a single experimental condition, that is important for inferring mechanisms, we examined whether a monotonic relationship exists between GC at the fMRI level and that at the neuronal level as a parameter is varied. The existence of such a relationship would allow us to interpret changes in fMRI Granger causality in terms of corresponding changes in neuronal Granger causality. Neural time series were obtained by simulating a bivariate AR model, which after HRF convolution became HRF-convolved time series, which after down-sampling to realistic TRs and addition of noise became fMRI time series (see Figure 1A). Three main results were found. First, GC from the HRF-convolved time series is a monotonic function of GC from the corresponding neural time series. Second, even with severe down-sampling and noise addition, monotonicity between fMRI GC and neural GC can still be reliably detected as a positive correlation. Third, HRF latency variability degrades the detectability of monotonicity, but a latency correction procedure significantly restores that detectability.

### Simulating fMRI Recordings

According to the linear transform model, fMRI signal is closely related to locally averaged neural activity such as local field potentials (LFPs) via HRF [28]. The subsequent steps, such as downsampling and noise addition, are also commonly practiced steps to convert neural signals to fMRI signals. Because it has been demonstrated that LFPs can be well described by autoregressive (AR) models [2], [7], [15], [36], our use of an AR model to generate the LFP signals is consistent with these findings and previous simulation studies of fMRI [11], [25]. The choice of 50 ms as temporal resolution in the model is reasonable for delays in large-scale brain networks which may vary from tens of milliseconds to hundreds of milliseconds. Single cell recordings in the macaque monkey [45] revealed a latency of 20 ms between neighboring regions in the visual hierarchy. Other intracranial recordings showed that neurons in inferior temporal cortex became activated 90–110 ms [46]–[48] after the stimulus onset and neural feedback could reach from hippocampus to inferior temporal visual cortex with latencies of 60–100 ms [49]. Similar ranges of neural delays have been consistently reported [50], [51]. Given that the human brain is significantly larger in size and more complex in structure than the monkey brain, neural delays in the human brain can be considerably longer. MEG recordings from humans have shown differences in peak onsets of 100∼200 ms between responses in the occipital cortex, the inferior frontal gyrus, and primary motor cortex [52]. Similar ranges of neural lags were repeatedly reported in other MEG- or EEG-studies [53]–[55].

### Linking fMRI GC with Neural GC

The goal of our work is different from some of the recent works on the applicability of Granger causality to fMRI data. Rather than focusing on detecting network configuration (e.g., whether there is a link from X to Y) [11], [18], [25], we examine whether there exists a relationship between fMRI GC and neural GC under variations of certain experimental conditions, with different experimental conditions being modeled by different coupling strengths between nodes of a network. This problem is relevant in that (1) over time scales resolvable by fMRI, the interactions in large-scale brain networks triggered by cognitive paradigms are likely to be bidirectional [40] and (2) in cognitive neuroscience, mechanisms of higher mental functions are inferred by assessing changes of dependent variables (e.g., GC) under changes of experimental conditions.

Detecting network configuration relies on statistical significance testing to ascertain whether a given GC is larger than zero or not. Past simulations have shown that such testing cannot prevent the detection of spurious Granger causality at the fMRI level, and that noise and downsampling can create nonzero GC when there is none at the neural level [11], [18], a fact we also found in our simulations. Our mathematical analysis in Appendix S1 demonstrates this rigorously. Thus observing GC change as a function of experimental parameters may be more meaningful in interpreting GC effects at the hemodynamic level in neural terms.

For the case of unidirectional coupling (Y→X is zero at neural level), the neural GC and the corresponding HRF-convolved GC for X→Y had nearly identical values, and the monotonicity relationship was clearly held with a slope close to 1 (Figure 1C). In addition, no spurious GC was found in this case (Figure 1D). These results suggest that convolution with HRF per se has no adverse effect on GC estimation. The two additional steps, down-sampling and noise addition, both essential for producing realistic fMRI time series, have the main negative impact on GC estimation. In agreement with the previous findings [11], along X→Y, the fMRI GC was reduced compared to the underlying neural GC (Figure 2A), and non-zero fMRI GC appeared along Y→X where the underlying neural GC is zero (Figure 2B). Despite these effects, the monotonicity between fMRI GC and neural GC along the same direction can still be reliably detected as a positive correlation, even in the presence of 20% measurement noise (SNR = 5) (Figures 2B and 2D), and fMRI Y→X and neural X→Y showed no systematic relationship. For bidirectional coupling, a case that is closer to reality in the brain but has not been considered in previous simulation studies, similar effects were found.

Using TPR, FPR and TDR to characterize the results, we found that although TPR decreased when more stringent correlation significance threshold was employed, the FPR was always significantly lower than TPR and approached zero faster (Figure 2D and Figure 3D). This means that even in low TPR situations, the detected monotonicity between fMRI GC and neural GC is an indicator of true monotonicity, and a more stringent significance threshold actually more strongly attenuates the chance for detecting false monotonicity, as reflected in enhanced TDR.

The numerical results above are further supported by analytical and numerical results presented in Appendix S1, where it is shown that although downsampling attenuates GC magnitude, there is a strict monotonic relationship between GC from the downsampled time series and that from the original time series, and HRF convolution preserves the monotonic relationship.

### Effects of Low Sampling Rate

Low sampling rate is a major reason for deteriorated Granger causality estimation at the fMRI level [56]. As expected, more severe down-sampling led to longer TR, which in turn led to lower TPR for detecting the monotonicity relationship. This is understandable because severe down-sampling makes signal transmission at faster time scales difficult to detect. However, even with large TR, the overall TDR remained at a high level of greater than 90% (Figure 4A), owing partly to the fact that FPR stayed below 5% for most of the TRs studied. Importantly, for TR between 1 s and 2 s, common values used in actual experimental recordings, both TDR and TPR were reasonably high. The reason that we could still reliably detect fMRI GC-neural GC monotonicity in the face of such downsampling rate may lie in the smoothing operation of the HRF. Following the HRF convolution, the model order of the resultant HRF-convolved neural series determined by BIC was around 15 to 17, which was much larger than the original model order of 1, indicating that the correlation structure is stretched in time, transforming faster neural dynamics to slower BOLD dynamics, which helps to preserve properties including monotonicity in the process. This effect is further demonstrated in Appendix S1. With the advent of technology, however, the adverse effects associated with low sampling rate may become less of a concern in the near future, because ultrafast sampling fMRI techniques are becoming increasingly available [41], [57] and our results show that high sampling rates improves monotonicity detection (Figures 1C and 1D, Figures 5F and 5G).

### Effects of Noise

The adverse effects of noise on Granger causality estimation have been studied in the past [23], [24], [58]. We tested how different levels of noise may affect monotonicity detection. When the noise level was lower than around 40% (SNR = 2.5), TDR and TPR were reasonably high while FPR were lower than 10% (Figure 3B). When noise level exceeded 40% (SNR = 2.5), both TDR and TPR began to decrease, and FPR increased. According to previous research, measurement noise around 20% (SNR = 5) seems to be a realistic noise level in actual fMRI recordings [11], [25]. In task experiments, when stronger variations evoked by task events are included, the proportion of measurement noise in the fMRI signal may be even lower.

### Effects of HRF Latency Variability

Different brain regions may have different hemodynamic response profiles [59]. If the HRF latency of region X is shorter than that of region Y, then detection of monotonicity between fMRI X→Y and neural X→Y is facilitated, while fMRI Y→X causality can become spurious. Based on findings that the HRF peak latency in cerebral cortex varies unsystematically within an individual [25], [38], [40], [59], in our simulation, the HRF latency difference between regions X and Y followed a normal distribution with zero mean across 100 experiments. By varying the standard deviation of the normal distribution, we found that although HRF latency difference degraded the detection of the underlying monotonicity by increasing FPR (Figures 5B and 5C), TDR remained in a range of 70%–80% when TR = 2 s (Figure 5F), and for much faster sampling rate of TR = 50 ms, it improved to over 90% (Figure 5G). The TDR, TPR and FPR curves in Figure 5C show that it is possible to obtain more reliable monotonicity detections by applying statistical criteria that more severely attenuate FPR. These results are in line with the finding that when HRF latency difference is unlikely to be systematic, the detected group level fMRI GC can reliably reflect neural influences [25]. However, when systematic HRF latency difference cannot be excluded, caution needs to be exercised in evaluating and interpreting the GC results [22].

A possible remedy for HRF latency induced deterioration of GC estimation is to estimate HRF latency and correct for it. We show that if HRF latency can be determined experimentally, then with latency correction [38], our ability to detect fMRI GC and neural GC monotonicity can be significantly improved (Figures 5D and 5E). TDR after latency correction stayed above 90% for the range of standard deviations of the latency difference tested (Figures 5F and 5G). There are a number of methods to estimate the regional HRF, such as selective averaging, window averaging, least-squares estimation, GLM fitting, Tikhonov regularization, and Bayesian estimation [39]–[41], [59]–[65]. These methods have shown their efficacy in estimating the regional HRF in the context of investigating the task activation of various brain areas. On the other hand, when dealing with global fMRI mapping performed voxel by voxel, previous studies have proposed the use of cerebrovascular response data to normalize or calibrate BOLD maps in order to reduce fMRI variability among brain areas in both within-subject analysis and cross-subject analysis [66]. Methods introduced for this purpose include the CO^2^ inhalation method and the breath holding (BH) method [38], [66]–[69]. Using the BH method Chang et al. [38] applied latency correction to improve Granger causality estimation.

### The “Third Variable” Problem

In the current simulation study we only considered pairwise GC. In the real brain the causal interactions between two brain areas may be mediated by a third brain area. Identifying and accounting for this “third variable” is therefore important in figuring out how information is routed in brain circuits. Conditional Granger causality is one way to deal with this problem [2], [3], [70], [71]. Examining fMRI-GC’s neural interpretability when the third variable is taken into account is one of the future research directions. However, as the below review of two recent empirical fMRI-GC studies demonstrate [43], [44], even pairwise GC can generate meaningful insights into cortical network operations.

### Empirical Validation

Real world neural time series are far more complex than that generated by AR (1). The assumed steps in transforming neural time series to fMRI time series, including HRF convolution, down-sampling and noise addition, are only an approximation of the actual physiological cardio-neural coupling [11], [28]. Ultimately, whether or not GC can be effectively applied to fMRI data has to be settled empirically, and a strong theoretical framework is crucial in such validation studies. Correlating Granger causality with various experimental parameters such as reaction time [26], [72] and BOLD level [73] has proven to be a fruitful approach.

In a recent paper we considered the behavioral consequences of the interaction between the dorsal attention network (DAN) and the ventral attention network (VAN) [43]. Extensive neural imaging and lesion evidence suggest that DAN is engaged in top-down attentional control and enables sensory motor processing whereas VAN mediates bottom-up attention reorienting [74], [75]. This theory led to the hypotheses that stronger causal influence from DAN to the VAN should be associated with enhanced behavioral performance, whereas stronger causal influence in the opposite direction should be associated with degraded behavioral performance [74]. This hypothesis was supported by a Granger causality analysis of the fMRI data in which the systematic relation between GC and performance parameters plays a critical role [43].

In another recent paper, applying the same approach, we investigated the interactions between the so-called task control network (TCN) and the default mode network (DMN). It has been hypothesized that TCN exerts top-down control by issuing signals to regulate activities in different brain areas to facilitate task performance [76]. In contrast, DMN, known to mediate self-referential processes, can be thought of as a source of internal noise when performing tasks requiring externally directed attention. These considerations predict that strong TCN→DMN should be associated with enhanced behavioral performance, whereas strong DMN→TCN should be associated with degraded behavioral performance. This hypothesis was again supported by correlating fMRI GC and behavioral performance [44].

## Supporting Information

Appendix S1 **Mathematical analysis of effect of downsampling and HRF convolution on Granger causality.** This Appendix has two objectives. First, we provide an analytic treatment of the effect of downsampling on Granger causality for an autoregressive (AR) model of order 1 (AR(1)), to further demonstrate that Granger causality from the downsampled signal is an monotonic function of Granger causality from the original signal. Second, we show that HRF convolution preserves and in fact enhances the monotonic relationship.
(DOCX)

## References

[1] Granger C (1969) Investigating causal relations by econometric models and crossspectral methods. Econometrica 37(3): 424–438.

[2] Ding M, Chen Y, Bressler S (2006) Granger causality: Basic theory and application to neuroscience. In: Schelter S, Winterhalder N, Timmer J, editors. Handbook of Time Series Analysis. Berlin Wiley-VCH. p. 437–460.

[3] Zhou Z, Ding M, Chen Y, Wright P, Lu Z, et al (2009) Detecting directional influence in fMRI connectivity analysis using PCA based Granger causality. Brain Res. 1289: 22–29. 10.1016/j.brainres.2009.06.096 PMC2730503 19595679

[4] Bollimunta A, Mo J, Schroeder CE, Ding M (2011) Neuronal mechanisms and attentional modulation of corticothalamic α oscillations. J Neurosci. 31(13): 4935–4943. 10.1523/JNEUROSCI.5580-10.2011 PMC3505610 21451032

[5] Bressler SL, Seth AK (2011) Wiener-Granger causality: a well established methodology. Neuroimage. 58(2): 323–329. 10.1016/j.neuroimage.2010.02.059 20202481

[6] Brovelli A, Ding M, Ledberg A, Chen Y, Nakamura R, et al (2004) Beta oscillations in a large-scale sensorimotor cortical network: Directional influences revealed by Granger causality. Proc Natl Acad Sci. 101: 9849–9854. 10.1073/pnas.0308538101 PMC470781 15210971

[7] Bollimunta A, Chen Y, Schroeder CE, Ding M (2008) Neuronal mechanisms of cortical alpha oscillations in awake-behaving macaques. J Neurosci. 28(40): 9976–9988. 10.1523/JNEUROSCI.2699-08.2008 PMC2692971 18829955

[8] Curtis CE, Sun FT, Miller LM, D'Esposito M (2005) Coherence between fMRI time-series distinguishes two spatial working memory networks. Neuroimage. 26(1): 177–183. 10.1016/j.neuroimage.2005.01.040 15862217

[9] Gelfand I, Yaglom A (1959) Calculation of the amount of information about a random function contained in another such function. Amer Math Soc Transl Ser. 2(12): 99.

[10] Wen X, Mo J, Ding M (2012) Exploring resting-state functional connectivity with total interdependence. Neuroimage. 60(2): 1587–1595. 10.1016/j.neuroimage.2012.01.079 PMC3516187 22289806

[11] Roebroeck A, Formisano E, Goebel R (2005) Mapping directed influence over the brain using Granger causality and fMRI. Neuroimage 25(1): 230–242. 15734358 10.1016/j.neuroimage.2004.11.017

[12] Roebroeck A, Formisano E, Goebel R (2011) The identification of interacting networks in the brain using fMRI: Model selection, causality and deconvolution. Neuroimage 58(2): 296–302. 19786106 10.1016/j.neuroimage.2009.09.036

[13] David O, Guillemain I, Saillet S, Reyt S, Deransart C, et al (2008) Identifying neural drivers with functional MRI: an electrophysiological validation. PLoS Biol 6(12): 2683–2697. 19108604 10.1371/journal.pbio.0060315 PMC2605917

[14] Witt ST, Meyerand ME (2009) The Effects of Computational Method, Data Modeling, and TR on Effective Connectivity Results. Brain Imaging Behav 3(2): 220–231. 19714255 10.1007/s11682-009-9064-5 PMC2731943

[15] Deshpande G, Sathian K, Hu X (2010) Effect of hemodynamic variability on Granger causality analysis of fMRI. Neuroimage 52(3): 884–896. 20004248 10.1016/j.neuroimage.2009.11.060 PMC3098126

[16] Florin E, Gross J, Pfeifer J, Fink GR, Timmermann L (2010) The effect of filtering on Granger causality based multivariate causality measures. Neuroimage 50(2): 577–588. 20026279 10.1016/j.neuroimage.2009.12.050

[17] Friston K (2011) Dynamic causal modeling and Granger causality Comments on: the identification of interacting networks in the brain using fMRI: model selection, causality and deconvolution. Neuroimage 58(2): 303–5; author reply 10–11. 10.1016/j.neuroimage.2009.09.031 PMC3183826 19770049

[18] Smith SM, Miller KL, Salimi-Korshidi G, Webster M, Beckmann CF, et al (2011) Network modelling methods for FMRI. Neuroimage 54(2): 875–891. 20817103 10.1016/j.neuroimage.2010.08.063

[19] David O (2011) fMRI connectivity, meaning and empiricism Comments on: Roebroeck et al. The identification of interacting networks in the brain using fMRI: model selection, causality and deconvolution. Neuroimage 58(2): 306–9; author reply 10–11. 10.1016/j.neuroimage.2009.09.073 19892020

[20] Friston K, Moran R, Seth AK (2013) Analysing connectivity with Granger causality and dynamic causal modelling. Curr Opin Neurobiol 23(2): 172–178. 23265964 10.1016/j.conb.2012.11.010 PMC3925802

[21] Aguirre GK, Zarahn E, D'esposito M (1998) The variability of human, BOLD hemodynamic responses. Neuroimage 8(4): 360–369. 9811554 10.1006/nimg.1998.0369

[22] Smith SM, Bandettini PA, Miller KL, Behrens TEJ, Friston KJ, et al (2012) The danger of systematic bias in group-level FMRI-lag-based causality estimation. Neuroimage 59(2): 1228–1229. 21867760 10.1016/j.neuroimage.2011.08.015

[23] Nalatore H, Ding M, Rangarajan G (2007) Mitigating the effects of measurement noise on Granger causality. Phys Rev E Stat Nonlin Soft Matter Phys 75(3 Pt 1): 031123. 10.1103/PhysRevE.75.031123 17500684

[24] Nalatore H, Ding M, Rangarajan G (2009) Denoising neural data with state-space smoothing: method and application. J Neurosci Methods 179(1): 131–141. 19428519 10.1016/j.jneumeth.2009.01.013 PMC2680758

[25] Schippers MB, Renken R, Keysers C (2011) The effect of intra- and inter-subject variability of hemodynamic responses on group level Granger causality analyses. Neuroimage 57(1): 22–36. 21316469 10.1016/j.neuroimage.2011.02.008

[26] Rypma B, Berger JS, Prabhakaran V, Bly BM, Kimberg DY, et al (2006) Neural correlates of cognitive efficiency. Neuroimage 33(3): 969–979. 17010646 10.1016/j.neuroimage.2006.05.065

[27] Miao X, Wu X, Li R, Chen K, Yao L (2011) Altered connectivity pattern of hubs in default-mode network with Alzheimer's disease: an Granger causality modeling approach. PLoS One 6(10): e25546. 22022410 10.1371/journal.pone.0025546 PMC3191142

[28] Boynton GM, Engel SA, Glover GH, Heeger DJ (1996) Linear systems analysis of functional magnetic resonance imaging in human V1. J Neurosci 16(13): 4207–4221. 8753882 10.1523/JNEUROSCI.16-13-04207.1996 PMC6579007

[29] Friston KJ, Fletcher P, Josephs O, Holmes A, Rugg MD, et al (1998) Event-related fMRI: characterizing differential responses. Neuroimage 7(1): 30–40. 9500830 10.1006/nimg.1997.0306

[30] Geweke J (1982) Measurement of linear dependence and feedback between multiple time series. J Am Stat Assoc 77(378): 304–313.

[31] Goebel R, Roebroeck A, Kim DS, Formisano E (2003) Investigating directed cortical interactions in time-resolved fMRI data using vector autoregressive modeling and Granger causality mapping. Magn Reson Imaging 21(10): 1251–1261. 14725933 10.1016/j.mri.2003.08.026

[32] Zang ZX, Yan CG, Dong ZY, Huang J, Zang YF (2012) Granger causality analysis implementation on MATLAB: a graphic user interface toolkit for fMRI data processing. J Neurosci Methods 203(2): 418–426. 22020117 10.1016/j.jneumeth.2011.10.006

[33] Hu S, Dai G, Worrell GA, Dai Q, Liang H (2011) Causality analysis of neural connectivity: critical examination of existing methods and advances of new methods. IEEE Trans Neural Netw 22(6): 829–844. 21511564 10.1109/TNN.2011.2123917 PMC3281296

[34] Hu S, Liang H (2012) Causality analysis of neural connectivity: New tool and limitations of spectralGranger causality. Neurocomputing 76: 44–47.

[35] Schwarz G (1978) Estimating the dimension of a model. Annals of Statistics 6(2): 461–464.

[36] Bressler SL, Tang W, Sylvester CM, Shulman GL, Corbetta M (2008) Top-down control of human visual cortex by frontal and parietal cortex in anticipatory visual spatial attention. J Neurosci 28(40): 10056–61. 18829963 10.1523/JNEUROSCI.1776-08.2008 PMC2583122

[37] Hamilton JP, Chen G, Thomason ME, Schwartz ME, Gotlib IH (2011) Investigating neural primacy in Major Depressive Disorder: multivariate Granger causality analysis of resting-state fMRI time-series data. Mol Psychiatry 16(7): 763–772. 20479758 10.1038/mp.2010.46 PMC2925061

[38] Chang C, Thomason ME, Glover GH (2008) Mapping and correction of vascular hemodynamic latency in the BOLD signal. Neuroimage 43(1): 90–102. 18656545 10.1016/j.neuroimage.2008.06.030 PMC2587338

[39] Dale AM, Buckner RL (1997) Selective averaging of rapidly presented individual trials using fMRI. Hum Brain Mapp 5(5): 329–340. 20408237 10.1002/(SICI)1097-0193(1997)5:5<329::AID-HBM1>3.0.CO;2-5

[40] Steffener J, Tabert M, Reuben A, Stern Y (2010) Investigating hemodynamic response variability at the group level using basis functions. Neuroimage 49(3): 2113–2122. 19913625 10.1016/j.neuroimage.2009.11.014 PMC2818488

[41] Katwal SB, Gore JC, Gatenby JC, Rogers BP (2012) Measuring relative timings of brain activities using fMRI. Neuroimage 66C: 436–448. 10.1016/j.neuroimage.2012.10.052 PMC3593774 23110880

[42] Menon RS, Luknowsky DC, Gati JS (1998) Mental chronometry using latency-resolved functional MRI. Proc Natl Acad Sci U S A 95(18): 10902–10907. 9724802 10.1073/pnas.95.18.10902 PMC27993

[43] Wen X, Yao L, Liu Y, Ding M (2012) Causal interactions in attention networks predict behavioral performance. J Neurosci 32(4): 1284–1292. 22279213 10.1523/JNEUROSCI.2817-11.2012 PMC6796284

[44] Wen X, Liu Y, Yao L, Ding M (2013) Top-down regulation of default mode activity in spatial visual attention. J Neurosci 33(15): 6444–6453. 23575842 10.1523/JNEUROSCI.4939-12.2013 PMC3670184

[45] Schmolesky MT, Wang Y, Pu M, Leventhal AG (2000) Degradation of stimulus selectivity of visual cortical cells in senescent rhesus monkeys. Nat Neurosci 3(4): 384–390. 10725929 10.1038/73957

[46] Rolls E (1992) Neurophysiology and functions of the primate amygdala. In: Aggleton J, editor. The amygdaleThe amygdale. New York: Wiley; p. 143–165.

[47] Rolls ET (2000) Memory systems in the brain. Annu Rev Psychol 51: 599–630. 10751982 10.1146/annurev.psych.51.1.599

[48] Rolls ET (2000) Functions of the primate temporal lobe cortical visual areas in invariant visual object and face recognition. Neuron 27(2): 205–218. 10985342 10.1016/s0896-6273(00)00030-1

[49] Rolls ET (2000) Hippocampo-cortical and cortico-cortical backprojections. Hippocampus 10(4): 380–388. 10985277 10.1002/1098-1063(2000)10:4<380::AID-HIPO4>3.0.CO;2-0

[50] Nakamura K, Matsumoto K, Mikami A, Kubota K (1994) Visual response properties of single neurons in the temporal pole of behaving monkeys. J Neurophysiol 71(3): 1206–1221. 8201413 10.1152/jn.1994.71.3.1206

[51] Lamme VA, Roelfsema PR (2000) The distinct modes of vision offered by feedforward and recurrent processing. Trends Neurosci 23(11): 571–579. 11074267 10.1016/s0166-2236(00)01657-x

[52] Nishitani N, Hari R (2002) Viewing lip forms: cortical dynamics. Neuron 36(6): 1211–1220. 12495633 10.1016/s0896-6273(02)01089-9

[53] Evdokimidis I, Smyrnis N, Constantinidis TS, Gourtzelidis P, Papageorgiou C (2001) Frontal-parietal activation differences observed before the execution of remembered saccades: an event-related potentials study. Brain Res Cogn Brain Res 12(1): 89–99. 11489612 10.1016/s0926-6410(01)00037-4

[54] McDowell JE, Kissler JM, Berg P, Dyckman KA, Gao Y, et al (2005) Electroencephalography/magnetoencephalography study of cortical activities preceding prosaccades and antisaccades. Neuroreport 16(7): 663–668. 15858402 10.1097/00001756-200505120-00002

[55] Sestieri C, Pizzella V, Cianflone F, Luca Romani G, Corbetta M (2007) Sequential activation of human oculomotor centers during planning of visually-guided eye movements: a combined fMRI-MEG study. Front Hum Neurosci 1: 1. 18958215 10.3389/neuro.09.001.2007 PMC2525985

[56] Seth AK, Chorley P, Barnett LC (2013) Granger causality analysis of fMRI BOLD signals is invariant to hemodynamic convolution but not downsampling. Neuroimage 65: 540–555. 23036449 10.1016/j.neuroimage.2012.09.049

[57] Feinberg DA, Moeller S, Smith SM, Auerbach E, Ramanna S, et al (2010) Multiplexed echo planar imaging for sub-second whole brain FMRI and fast diffusion imaging. PLoS One 5(12): e15710. 21187930 10.1371/journal.pone.0015710 PMC3004955

[58] Rogers BP, Katwal SB, Morgan VL, Asplund CL, Gore JC (2010) Functional MRI and multivariate autoregressive models. Magn Reson Imaging 28(8): 1058–1065. 20444566 10.1016/j.mri.2010.03.002 PMC2940955

[59] Handwerker DA, Ollinger JM, D'Esposito M (2004) Variation of BOLD hemodynamic responses across subjects and brain regions and their effects on statistical analyses. Neuroimage 21(4): 1639–1651. 15050587 10.1016/j.neuroimage.2003.11.029

[60] Wagner AD, Schacter DL, Rotte M, Koutstaal W, Maril A, et al (1998) Building memories: remembering and forgetting of verbal experiences as predicted by brain activity. Science 281(5380): 1188–1191. 9712582 10.1126/science.281.5380.1188

[61] Marrelec G, Benali H, Ciuciu P, Pélégrini-Issac M, Poline JB (2003) Robust Bayesian estimation of the hemodynamic response function in event-related BOLD fMRI using basic physiological information. Hum Brain Mapp 19(1): 1–17. 12731100 10.1002/hbm.10100 PMC6871990

[62] Wager TD, Keller MC, Lacey SC, Jonides J (2005) Increased sensitivity in neuroimaging analyses using robust regression. Neuroimage 26(1): 99–113. 15862210 10.1016/j.neuroimage.2005.01.011

[63] Casanova R, Ryali S, Serences J, Yang L, Kraft R, et al (2008) The impact of temporal regularization on estimates of the BOLD hemodynamic response function: a comparative analysis. Neuroimage 40(4): 1606–1618. 18329292 10.1016/j.neuroimage.2008.01.011 PMC2432527

[64] Kay KN, David SV, Prenger RJ, Hansen KA, Gallant JL (2008) Modeling low-frequency fluctuation and hemodynamic response timecourse in event-related fMRI. Hum Brain Mapp 29(2): 142–156. 17394212 10.1002/hbm.20379 PMC6871156

[65] Luchtmann M, Jachau K, Tempelmann C, Bernarding J (2010) Alcohol induced region-dependent alterations of hemodynamic response: implications for the statistical interpretation of pharmacological fMRI studies. Exp Brain Res 204(1): 1–10. 20502888 10.1007/s00221-010-2277-4 PMC2885301

[66] Magon S, Basso G, Farace P, Ricciardi GK, Beltramello A, et al (2009) Reproducibility of BOLD signal change induced by breath holding. Neuroimage 45(3): 702–712. 19211035 10.1016/j.neuroimage.2008.12.059

[67] Bandettini PA, Wong EC (1997) A hypercapnia-based normalization method for improved spatial localization of human brain activation with fMRI. NMR Biomed 10(4–5): 197–203. 9430348 10.1002/(sici)1099-1492(199706/08)10:4/5<197::aid-nbm466>3.0.co;2-s

[68] Davis TL, Kwong KK, Weisskoff RM, Rosen BR (1998) Calibrated functional MRI: mapping the dynamics of oxidative metabolism. Proc Natl Acad Sci U S A 95(4): 1834–1839. 9465103 10.1073/pnas.95.4.1834 PMC19199

[69] Cohen ER, Rostrup E, Sidaros K, Lund TE, Paulson OB, et al (2004) Hypercapnic normalization of BOLD fMRI: comparison across field strengths and pulse sequences. Neuroimage 23(2): 613–624. 15488411 10.1016/j.neuroimage.2004.06.021

[70] Chen Y, Bressler SL, Ding M (2006) Frequency decomposition of conditional Granger causality and application to multivariate neural field potential data. J Neurosci Methods 150(2): 228–37. 16099512 10.1016/j.jneumeth.2005.06.011

[71] Shokri-Kojori E, Motes MA, Rypma B, Krawczyk DC (2012) The network architecture of cortical processing in visuo-spatial reasoning. Sci Rep 2: 411. 22624092 10.1038/srep00411 PMC3355370

[72] Biswal BB, Eldreth DA, Motes MA, Rypma B (2010) Task-dependent individual differences in prefrontal connectivity. Cereb Cortex 20(9): 2188–2197. 20064942 10.1093/cercor/bhp284 PMC2923215

[73] Jiao Q, Lu G, Zhang Z, Zhong Y, Wang Z, et al (2011) Granger causal influence predicts BOLD activity levels in the default mode network. Hum Brain Mapp 32(1): 154–161. 21157880 10.1002/hbm.21065 PMC6870036

[74] Corbetta M, Shulman GL (2002) Control of goal-directed and stimulus-driven attention in the brain. Nat Rev Neurosci 3(3): 201–215. 11994752 10.1038/nrn755

[75] Corbetta M, Patel G, Shulman GL (2008) The reorienting system of the human brain: from environment to theory of mind. Neuron 58(3): 306–324. 18466742 10.1016/j.neuron.2008.04.017 PMC2441869

[76] Dosenbach NU, Visscher KM, Palmer ED, Miezin FM, Wenger KK, et al (2006) A core system for the implementation of task sets. Neuron 50(5): 799–812. 16731517 10.1016/j.neuron.2006.04.031 PMC3621133

