# Supplementary material for: Is Granger Causality a Viable Technique for Analyzing fMRI Data?
Source: PLoS One. 2013 Jul 4;8(7):e67428. doi: 10.1371/journal.pone.0067428 (PMC3701552; doi:10.1371/journal.pone.0067428)
Supplement: Appendix S1 — Mathematical analysis of effect of downsampling and HRF convolution on Granger causality. This Appendix has two objectives. First, we provide an analytic treatment of the effect of downsampling on Granger causality for an autoregressive (AR) model of order 1 (AR(1)), to further demonstrate that Granger causality from the downsampled signal is an monotonic function of Granger causality from the original signal. Second, we show that HRF convolution preserves and in fact enhances the monotonic relationship. (DOCX) [file pone.0067428.s001.docx]

**Appendix S1**

This Appendix has two objectives. First, we provide an analytic treatment of the effect of downsampling on Granger causality for an autoregressive (AR) model of order 1 (AR(1)), to further demonstrate that Granger causality from the downsampled signal is an monotonic function of Granger causality from the original signal. Second, we show that HRF convolution preserves and in fact enhances the monotonic relationship.

Consider the following AR(1) process where the coupling is unidirectional:

$$\begin{matrix} \mathbf{w}_{t}=\mathbf{Aw}_{t-1}+\mathbf{u}_{t}; \\ \mathbf{w}_{t}=\left(\begin{matrix} X_{t} \\ Y_{t} \end{matrix}\right), \mathbf{A}=\left(\begin{matrix} a & 0 \\ c & d \end{matrix}\right), \mathbf{u}_{t}=\left(\begin{matrix} \varepsilon_{t} \\ \eta_{t} \end{matrix}\right), t=1,2,3,... \end{matrix}$$

where $\mathbf{u}_{t}$ is a vector white noise process with zero mean and covariance matrix

$\mathbf{\Sigma}=\left(\begin{matrix} \Sigma& 0 \\ 0 & \Gamma\end{matrix}\right)$ ,

which, for simplicity, is assumed to be diagonal. In this case, an explicit expression for Granger causality $I_{X\to Y}(f)$ from *X* to *Y* at frequency $f$ is given by

$$I_{X\to Y}(f)=\ln\left[1+\frac{c^{2}\Sigma}{\Gamma(1+a^{2}-2a\cos\frac{2\pi f}{f_{s}})}\right]$$

where $f_{s}$ is the sampling frequency, and $I_{Y\to X}(f)=0$for all frequencies. Granger causality in the time domain is obtained by integrating the above expression over all frequencies in the interval$[-f_{s},f_{s}]$.

When the signal generated by the above AR process is downsampled by a factor of 2, data at $t=2,4,...$are recorded, and $\mathbf{w}_{t}$ need to be expressed in terms of $\mathbf{w}_{t-2}$. By iterating the above AR(1) model we obtain:

$\mathbf{w}_{t}=\mathbf{A}[\mathbf{Aw}_{t-2}+\mathbf{u}_{t-1}]+\mathbf{u}_{t}=\mathbf{A}^{2}\mathbf{w}_{t-2}+\mathbf{Au}_{t-1}+\mathbf{u}_{t}$.

Let $\mathbf{u}'_{t}=\mathbf{Au}_{t-1}+\mathbf{u}_{t}$. It can be easily shown that $\mathbf{u}'_{t}$ is again a zero mean white noise process for $t=2,4,...$with contemporaneous covariance matrix

$\mathbf{\Sigma}'=\mathbf{A\Sigma A}^{T}+\mathbf{\Sigma}$, 

where *T* denotes matrix transposition. Thus

$$\mathbf{w}_{t}=\mathbf{A}^{2}\mathbf{w}_{t-2}+\mathbf{u}'_{t}, t=2,4,...$$

is a valid AR process. Letting $t=2t'$ we have

$\mathbf{w}_{2t'}=\mathbf{A}^{2}\mathbf{w}_{t-2}+\mathbf{u}'_{t}, t=2,4,...$.

This can be considered as an AR(1) process $\mathbf{w}'_{t'}$ in $t'$:

$\mathbf{w}'_{t'}=\mathbf{A}^{2}\mathbf{w}'_{t'-1}+\mathbf{u}'_{t'},t'=1,2,...$,

with the proviso that the new sampling frequency $f_{s}'=f_{s}/2$. Putting everything together, we finally have the following new AR(1) process which models the downsampled signal:

$\mathbf{w}'_{t'}=\mathbf{A}'\mathbf{w}'_{t'-1}+\mathbf{\varepsilon}'_{t'}, t'=1,2,..., \mathbf{A}'=\mathbf{A}^{2}, \mathbf{\Sigma}'=\mathbf{A\Sigma A}^{T}+\mathbf{\Sigma}, f_{s}'=f_{s}/2$.

Note that

$$\mathbf{A}'=\left(\begin{matrix} a' & 0 \\ c' & d' \end{matrix}\right)$$

where $a'=a^{2}$, $c'=c(a+d)$, $d'=d^{2}$ and

$$\mathbf{\Sigma}'=\left(\begin{matrix} \Sigma' & \gamma' \\ \gamma' & \Gamma' \end{matrix}\right)$$

with $\Sigma'=a^{2}\Sigma$, $\gamma'=ac\Sigma$,$\Gamma'=c^{2}\Sigma+d^{2}\Gamma$. Since the above process is also an AR(1) process, following the standard procedure we obtain Granger causality for the downsampled signal to be

$I'_{X\to Y}(f)=\ln\left[1+\frac{c'^{2}(\Sigma'\Gamma'-\gamma'^{2})}{(1+a'^{2})\Gamma'^{2}-2a'c'\Gamma'\gamma'+c'^{2}\gamma'^{2}-2(a'\Gamma'-c'\gamma')\Gamma'\cos\frac{2\pi f}{f_{s}'}}\right]$;$I'_{Y\to X}(f)=0$.

Again, Granger causality in the time domain is obtained by integrating the above expressions over all frequencies in the interval $[-f_{s}',f_{s}']$.

In Figure S6A the analytically evaluated time domain Granger causality (GC) of X🡪Y is displayed as a function of $c$ for both the original and the downsampled signal (solid curves). Here we have set $a=0.3$, $d=0.5$, $\Sigma=\Gamma=0.5$, $f_{s}=$ 20 Hz. Although the magnitude of X🡪Y GC for the downsampled signal is smaller than that for the original signal, it varies monotonically as a function of $c$, just like X🡪Y GC for the original signal. This further implies that X🡪Y GC after downsampling is a monotonically increasing function of original X🡪Y GC as demonstrated in Figure S6B. Numerically computed X🡪Y GC from multiple realizations of the AR(1) process and its down-sampled version agrees very well with the analytical results. The above analysis can be generalized to the case of bidirectional coupling and to the case of downsampling by higher factors. The conclusions remain the same.


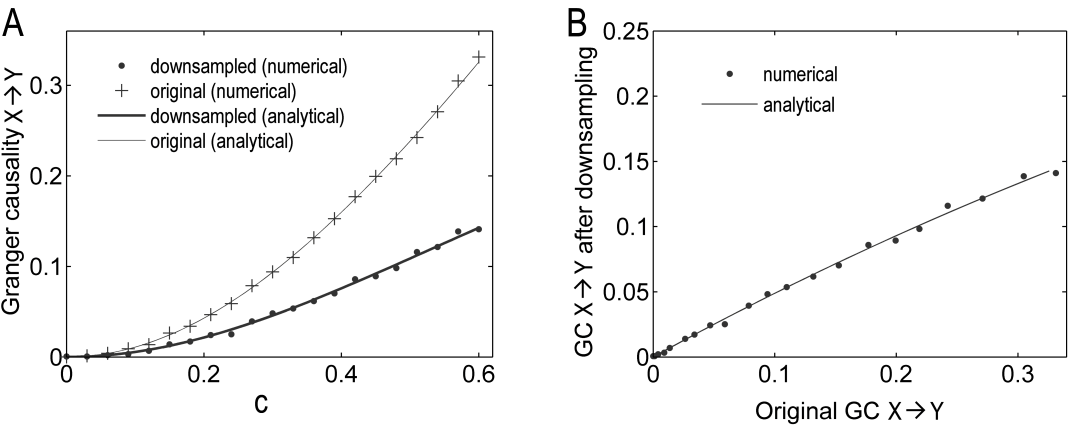


**Figure S6.** **Further analysis of the effect of downsampling.** A: Monotonic relationship between X🡪Y GC and AR coefficient *b*, before and after downsampling. B: Monotonic relationship between X🡪Y GC after downsampling and original X🡪Y GC.

Next, we consider convolution of the original signal by an HRF and then down-sample by a factor of 2. The analytical treatment of this case is complicated and beyond the scope of this appendix. The numerical results are displayed in Figure S7. We make three observations. First, convolution counteracts the reduction in magnitude of Granger causality caused by downsampling. This is to be expected since convolution by a slowly varying function such as an HRF smoothens the signal and spreads information out over a larger time interval (in mathematical terms, the original AR(1) process was Markovian whereas the convolved process has ‘long’ memory). Second, the variation of X🡪Y GC with $c$ is still monotonic even for the convolved and downsampled signal (Figure S7A), and X🡪Y GC after convolution and downsampling remains a monotonically increasing function of original X🡪Y GC. Third, although the analysis can be generalized to bi-directional coupling and lower sampling rate, the algebra becomes quite unwieldy. It is more difficult to extend it to multivariate processes [77].


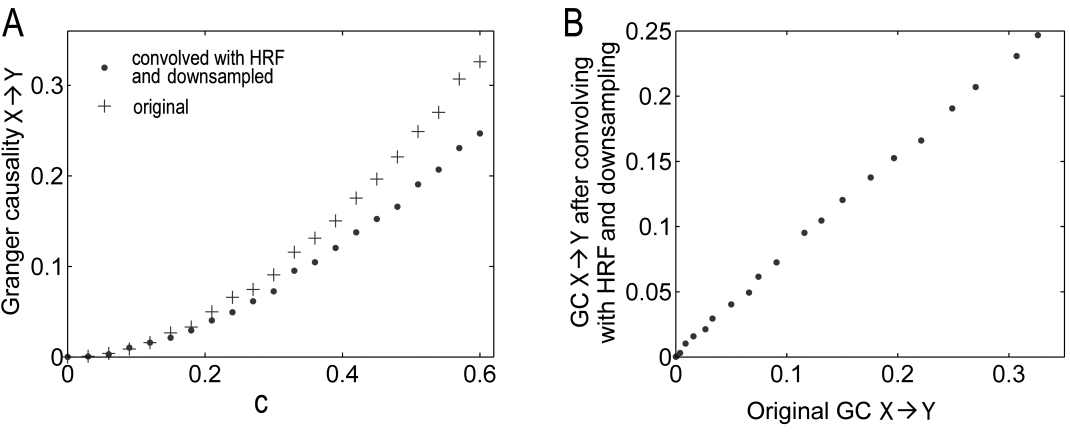


**Figure S7.** **Further analysis of the effect of HRF convolution.** A: Monotonic relationship between HRF-convolved X🡪Y GC and AR coefficient *b*, before and after HRF-convolution and downsampling. B: Monotonic relationship between X🡪Y GC after HRF convolution and downsampling and original X🡪Y GC.

**Reference**

1. Valdes-Sosa PA, Roebroeck A, Daunizeau J, Friston K (2011) Effective connectivity: influence, causality and biophysical modeling. Neuroimage 58(2):339-361.
